# Development and Validation of an m6A RNA Methylation Regulators-Based Signature for Predicting the Prognosis of Adrenocortical Carcinoma

**DOI:** 10.3389/fendo.2021.568397

**Published:** 2021-02-22

**Authors:** Chengquan Shen, Jing Liu, Xiaokun Yang, Wei Jiao, Yonghua Wang

**Affiliations:** ^1^ Department of Urology, The Affiliated Hospital of Qingdao University, Qingdao, China; ^2^ Department of Research Management and International Cooperation, The Affiliated Hospital of Qingdao University, Qingdao, China

**Keywords:** adrenocortical carcinoma, m6A RNA methylation regulators, prognosis, signature, The Cancer Genome Atlas

## Abstract

**Background:**

Adrenocortical carcinoma (ACC) is an aggressive and rare neoplasm that originates from the cortex of the adrenal gland. N6-methyladenosine (m6A) RNA methylation, the most common form of mRNA modification, has been reported to be correlated with the occurrence and development of the malignant tumor. This study aims to identify the significance of m6A RNA methylation regulators in ACC and construct a m6A based signature to predict the prognosis of ACC patients.

**Materials and methods:**

RNA-seq data from The Cancer Genome Atlas (TCGA) database was used to identify the expression level of m6A RNA methylation regulators in ACC. An m6A based signature was further constructed and its prognostic and predictive values were assessed by survival analysis and nomogram.

**Results:**

11 m6A RNA regulators were differentially expressed in ACC and three m6A RNA regulators were finally selected in a signature to predict the prognosis of ACC patients. Survival analysis indicated that high risk scores were closely related to poor survival outcomes in ACC patients. Univariate and multivariate Cox regression analyses demonstrated that the m6A based signature was an independent prognostic factor for ACC patients. A nomogram with clinical factors and the m6A based signature was also constructed to superiorly predict the prognosis of ACC patients. The expression levels of m6A RNA methylation regulators, which were contained in the signature, were also verified in human ACC tissues and normal tissues by using vitro experiments.

**Conclusion:**

We identified and validated an m6A based signature, which can be used as an independent prognostic factor in evaluating the prognosis of ACC patients. Further clinical trials and experimental explorations are needed to confirm our observations and mechanisms underlying prognostic values of these m6A RNA methylation regulators in ACC.

## Introduction

Adrenocortical carcinoma (ACC) is a rare disease of the adrenal cortex with aggressive behavior, and only 16% to 47% of patients are still alive after five years (1). The significant progress in the treatment of ACC, the molecular mechanisms, drug resistance, and reliable prognostic biomarkers in ACC remain poorly understood. Over the past decade, methods in genomic study have multiplied our knowledge about gene expression, genetic, and epigenetic alterations at the pan-genomic level in ACC. This highlights the importance of genetic counseling to identify potential prognostic biomarkers in patients with ACC. N6-methyladenosine (m6A) is the most abundant internal modification of mRNAs in higher eukaryotes and mainly influences mRNA stability, translation efficiency, variable splicing, and localization by dynamic regulation of methyltransferases (“writers”), m6A-binding proteins (“readers”), and demethylases (“erasers”) (2–4). Previous studies also demonstrated that m6A RNA regulators were significantly associated with the prognosis, chemoresistance, and radioresistance of cancers, such as breast cancer, bladder cancer, gastric cancer, and pancreatic cancer (5–8). In addition, m6A RNA methylation regulators can promote the occurrence and progression of cancers by multiple signaling pathways, such as regulating the epithelial mesenchymal transition of cancer, Wnt/PI3K-Akt signaling, and modulating genes expression levels (9–11). However, the expression levels and prognostic values of m6A RNA methylation regulators in ACC have not been elucidated.

In the present study, we first utilized the transcriptome data from The Cancer Genome Atlas (TCGA) to identify the significance of 13 m6A RNA methylation regulators in ACC patients. We evaluated the interaction and correlation among m6A RNA methylation regulators and identified two clusters of ACC patients with different survival outcomes. Subsequently, an m6A based signature was further developed and was closely related to the prognosis and clinicopathologic characteristics of ACC patients. Furthermore, a nomogram was constructed to validate its predictive value in ACC.

## Materials and Methods

### Data Collection

RNA-sequencing (RNA-seq) expression profiles of the ACC cohort were obtained from TCGA official website (https://tcga‐data.nci.nih.gov/tcga/). A total of 92 patients were enrolled in our study and detailed clinical characteristics are described in **Table 1**. RNA-sequencing (RNA-seq) expression profiles of normal cohort contained 128 normal samples, which were download from the Genotype-Tissue Expression (GTEx) database (https://xenabrowser.net/datapages/). Thirteen widely recognized m6A RNA regulators were retrieved from published literature, including *METTL3* (methyltransferase-like 3), *METTL14* (methyltransferase-like 14), *WTAP* (Wilms tumor 1-associated protein), *KIAA1429* (VIRMA, vir like m6A methyltransferase associated), *RBM15* (RNA binding motif protein 15), *FTO* (fat mass and obesity-associated gene), *ALKBH5* (alkB homolog 5, RNA demethylase), *YTHDC1* (YTH domain containing 1), *YTHDC2* (YTH domain containing 2), *YTHDF1* (YTH N6-methyladenosine RNA binding protein 1), *YTHDF2* (YTH N6-methyladenosine RNA binding protein 2), *HNRNPC* (heterogeneous nuclear ribonucleoprotein C), and *ZC3H13* (zinc finger CCCH-type containing 13).

**Table 1:** TCGA ACC patient characteristics.

Clinical characteristics		Total (92)	%
Age		49 (14-83)	
Gender	Female	60	65.22
	Male	32	34.78
Grade	Unknown		
Stage	I	9	9.78
	II	44	47.83
	III	19	20.65
	IV	18	19.57
	Unknown	2	2.17
T	T1	9	9.78
	T2	49	53.26
	T3	11	11.96
	T4	21	22.83
	Unknown	2	2.17
N	N0	80	86.96
	N1	10	10.87
	Unknown	2	2.17
M	M0	72	78.26
	M1	18	19.57
	Unknown	2	2.17
Histological type	Myxoid type	1	1.09
	Oncocytic type	4	4.35
	Usual type	87	94.56
Invasion of tumor capsule	Absent	35	38.05
	Present	48	52.17
	Unknown	9	9.78
Mitotane therapy	No	35	38.04
	Yes	55	59.79
	Unknown	2	2.17
Radiation therapy	No	71	77.17
	Yes	18	19.57
	Unknown	3	3.26
Primary therapy outcome success	Complete remission	56	60.87
	Progression disease	21	22.83
	Stable disease	2	2.17
	Unknown	13	14.13

### Differential Expression Analysis of m6A RNA Methylation Regulators in ACC

The expression levels of 13 m6A RNA regulators between ACC and normal samples were measured using edgeR (version R 3.5.1, https://bioconductor.org/packages/release/bioc/) (12). *P* < 0.05 was recognized as the cut-off value. Heatmap and violin plot were used to display the expression levels of m6A RNA methylation regulators in ACC and normal samples.

### Consensus Clustering Analysis

To determine the relationship between the expression levels of m6A RNA regulators and the prognosis of ACC patients, we classified ACC patients into different groups by consensus clustering analysis with “ConsensusClusterPlus” in R. Kaplan-Meier method was performed to investigate the survival differences between two clusters.

### Construction and Evaluation of an m6A Based Signature

Univariate Cox regression analysis was performed using the R package “survival” to identify m6A RNA methylation regulators that were associated with the OS of ACC patients. A least absolute shrinkage and selection operator (LASSO) Cox regression method was adopted to establish an optimal m6A based signature for predicting the prognosis of ACC patients by using the R package called “glmnet” and the m6A based signature risk score = Σ(*β_i_*×Exp*_i_*) (*i* = the number of m6A RNA regulators) (13, 14). After calculating the risk scores of ACC patients, patients were further stratified into high-risk and low-risk groups according to the median risk score. Kaplan-Meier curves were used to evaluate the survival outcome differences between the high-risk and low-risk groups. The survival ROC package was used to conduct the receiver operating characteristic curve (ROC) to evaluate the prediction accuracy of the m6A based signature. Univariate and multivariate Cox regression analyses were used to explore prognostic values of the m6A based signature and various clinical characteristics. In addition, the Wilcoxon signed-rank test was applied to identify the relationship between the m6A based signature risk score and clinicopathological characteristics.

### Development of a Nomogram Based on the Signature Risk Score and Clinicopathological Characteristics

Clinical factors (age, gender, stage, T, N, M, invasion of tumor capsule, mitotane therapy, radiation therapy) and the m6A based signature risk score were used to construct a prognostic nomogram to assess the probability of 1-, 2-, and 3-year OS for ACC patients *via* the R package (https://cran.r-project.org/web/packages/rms/) (15).

### RNA Isolation and Reverse Transcription−Quantitative PCR

To further validate the expression levels of three m6A RNA regulators in our ACC tissues and normal tissues, RNA isolation and reverse transcription−quantitative PCR (RT-qPCR) were performed. The RNAprep pure FFPE kit (DP439, TIANGEN Biotech(Beijing)Co, Ltd, CHN) was used to extract the total RNA from tissue specimens based on the manufacturer’s instructions. For the detection of mRNA levels, the total RNA (500 ng) was transcribed into cDNA using a PrimeScript™ RT reagent kit (Perfect Real Time) (Takara, code no RR037A). All the primers were synthesized by Huada Gene (Beijing, China) and the sequences are shown in **Table 2**. The amplification of cDNAs was conducted with Roche LightCycler 480II real-time PCR detection system (Roche, Basel, Switzerland). Gene expression was normalized against GADPH and relative expression levels of *RBM15*, *FTO*, and *HNRNPC* were determined by the comparative threshold cycle (Ct) method using the formula 2−(ΔΔCt).

**Table 2:** Sequences of the primers used for real-time quantitative PCR.

Name of primer	Sequence of primer (5’ to 3’)
FTO-F	AATAGCCGCTGCTTGTGAGA
FTO-R	CAATGGCACAGCATCCTCAT
HNRNPC-F	AGAACCCGGGAGTAGGAGAC
HNRNPC-R	TCTCACAAAGCCGAAAACAA
RBM15-F	TCCCACCTTGTGAGTTCTCC
RBM15-R	GTCAGCGCCAAGTTTTCTCT

## Results

### Expression Patterns of 13 m6A RNA Regulators in ACC

**Figure 1:**
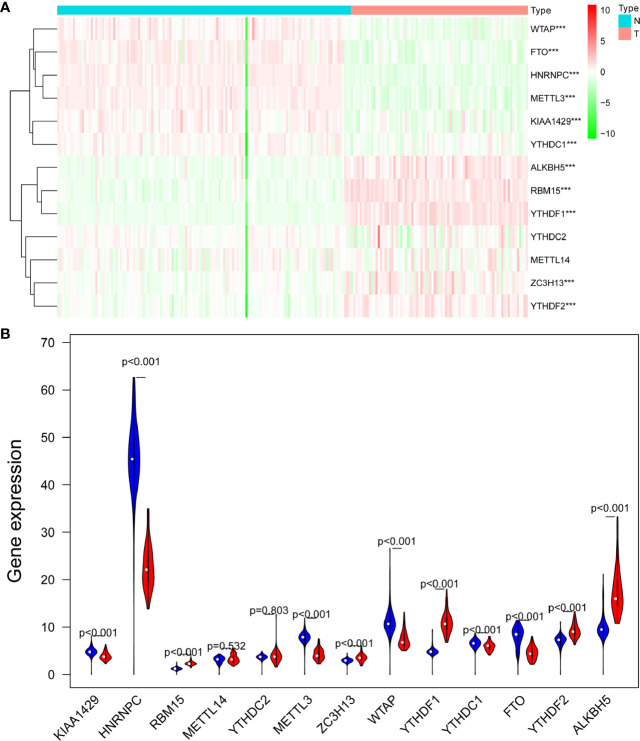
The expression levels of 13 m6A RNA methylation regulators between ACC and normal samples. **(A)** The heatmap was used to screen the expression levels of m6A RNA methylation regulators in each clinical sample. **(B)** The violin plot showed the significantly differentially expressed m6A RNA methylation regulators between ACC and the normal samples. ***p < 0.005.

A heatmap was generated to screen the expression levels of 13 m6A RNA regulators between ACC and normal samples. Red represents high expression, while green represents low expression. *KIAA1429* (*p* < 0.001), *HNRNPC* (*p* < 0.001), *RBM15* (*p* < 0.001), *METTL3* (*p* < 0.001), *ZC3H13* (*p* < 0.001), *WTAP* (*p* < 0.001), *YTHDF1* (*p* < 0.001), FTO (*p* < 0.001), *YTHDF2* (*p* < 0.001), and *ALKBH5* (*p* < 0.001) were differentially expressed in ACC samples (**Figure 1A**). Compared to normal tissue samples, *KIAA1429*, *HNRNPC*, *METTL3*, *WTAP*, *YTHDC1*, and *FTO* were down-regulated in ACC, and *RBM15*, *ZC3H13*, *YTHDF1*, *YTHDF2*, and *ALKBH5* were up-regulated in ACC (**Figure 1B**).

### Consensus Clustering of m6A RNA Methylation Regulators Identified Two Clusters of ACC Patients With Different Survival Outcomes

**Figure 2:**
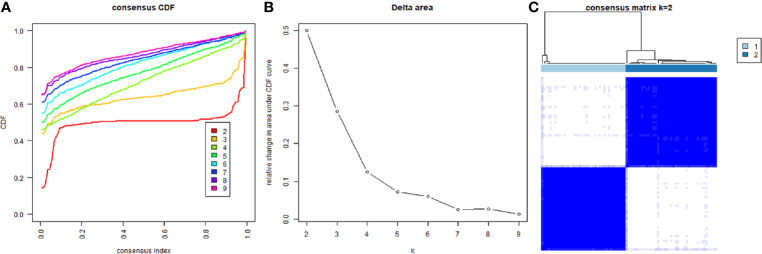
Divided ACC patients into different groups according to the expression of m6A RNA methylation regulators. **(A)** Consensus clustering cumulative distribution function CDF for k = 2 to 9. **(B)** Relative change in area under CDF curve for k = 2 to 9. **(C)** The TCGA ACC cohort was divided into two distinct clusters when k = 2.

**Figure 3:**
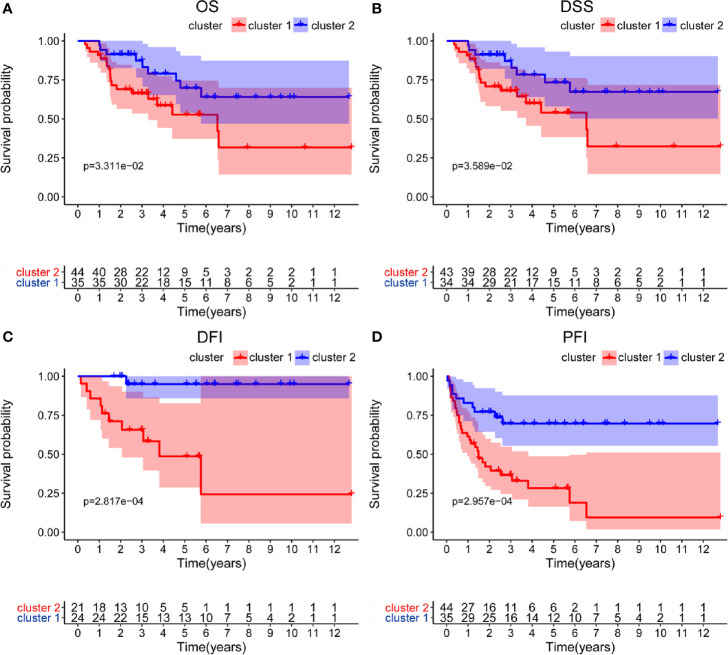
Differential survival outcomes of ACC patients in the two different clusters. **(A–D)** Survival analysis indicated that a significant shorter overall survival (OS), disease-specific survival (DSS), disease-free interval (DFI), and progression-free interval (PFI) were observed in ACC patients in cluster 2 than those in cluster 1.

Based on the expression similarity of m6A RNA regulators, k=2 was the most optimal approach to divide ACC patients into two clusters, namely cluster 1 and cluster 2 (**Figures 2A-C**). Compare with cluster 1, most m6A RNA methylation regulators were highly expressed in cluster 2 (**Supplementary 1**). Survival analysis indicated that a significantly shorter overall survival (OS) (*p* = 3.311e-02), disease-specific survival (DSS) (*p* = 3.589e-02), disease-free interval (DFI) (*p* = 2.817e-04), and progression-free interval (PFI) (*p* = 2.957e-04) were observed in ACC patients in cluster 2 compared to those in cluster 1 (**Figures 3A-D**).

### Identification of Survival-Related m6A RNA Regulators and Construction of a m6A Based Signature

**Figure 4:**
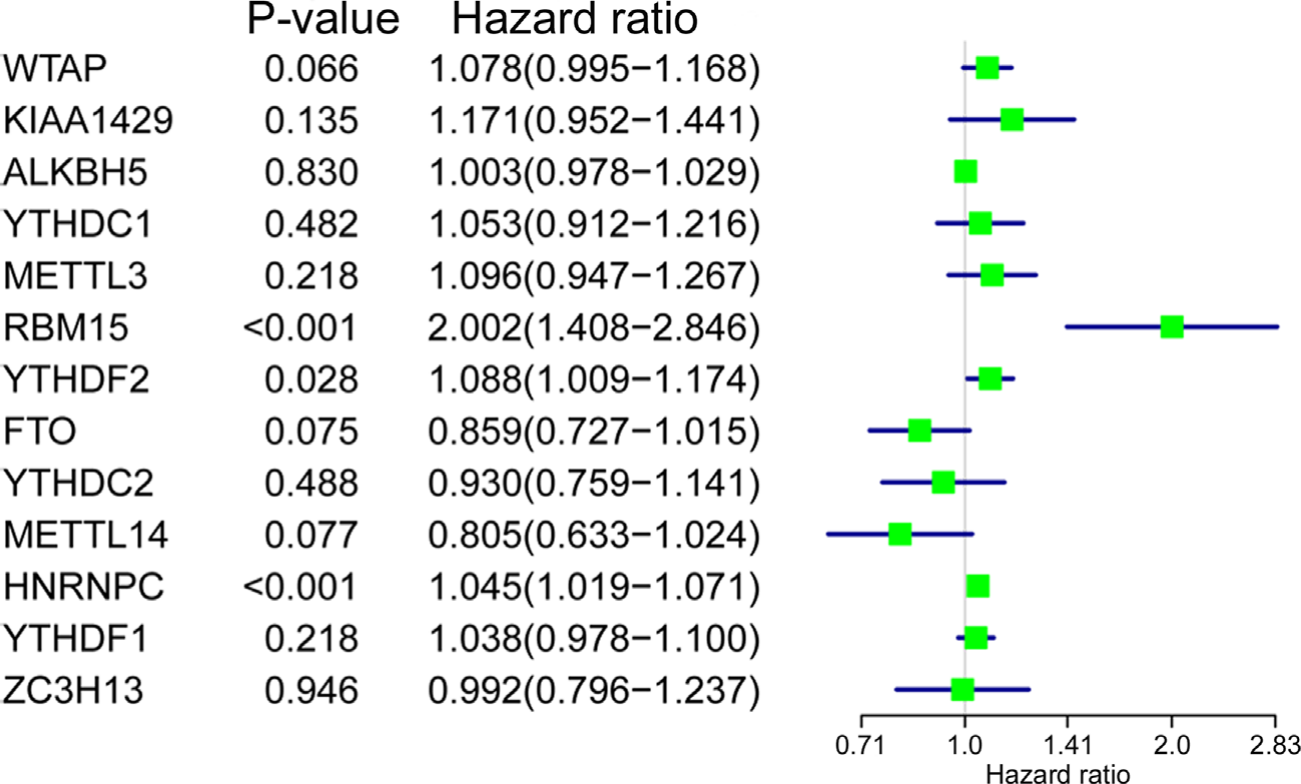
The relationship between m6A RNA methylation regulators and OS of ACC patients. Univariate Cox regression analysis showed that *RBM15*, *YTHDF2*, and *HNRNPC* are closely associated with the OS of BC patients (*p* < 0.05).

**Figure 5:**
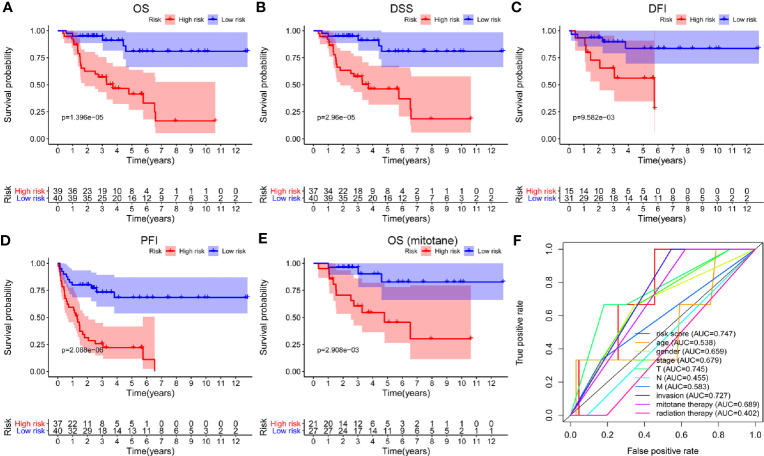
Survival analysis of the m6A based signature in ACC. **(A-D)** Kaplan-Meier curves revealed that the high-risk group had significantly shorter overall survival (OS), disease-specific survival (DSS), disease-free interval (DFI), and progression-free interval (PFI) compared with the low-risk group. **(E)** In addition, a high risk score also indicated poor OS in ACC patients who received mitotane therapy. **(F)** ROC curves showed that the area under the curves (AUCs) of risk score, age, gender, stage, T, N, M, invasion, mitotane therapy, and radiation therapy were 0.747, 0.538,0.659, 0.679,0.745, 0.455, 0.583, 0.727, 0.689, and 0.402.

Univariate Cox regression analysis was applied to identify survival-related m6A RNA regulators in ACC patients. The result demonstrated that *RBM15* (*p* < 0.001), *HNRNPC* (*p* < 0.001), and *YTHDF2* (*p* = 0.028) were significantly related to OS and these three m6A regulators were risk genes with HR larger than 1 (**Figure 4**). Subsequently, LASSO Cox regression analysis was performed to construct an m6A based signature that contained three m6A RNA regulators, including *RBM15*, *HNRNPC*, and *FTO*. We further calculated the risk score for ACC patients as follows: risk score = (0.3070 × expression value of *RBM15*) + (0.0235 × expression value of *HNRNPC*) + (-0.0673 × expression value of *FTO*), and divided ACC patients into high-risk and low-risk groups following the cut-off of the median risk score. Survival analysis revealed that the high-risk group had shorter OS (*p* = 1.396e-05), DSS (*p* = 2.96e-05), DFI (*p* = 9.582e-03), and PFI (*p* = 2.088e-06) when compared with the low-risk group (**Figures 5A-D**). In addition, high risk scores also indicate poor OS in ACC patients, which received mitotane therapy (*p* = 2.908e-03, **Figure 5E**). Furthermore, ROC curves were used to validate the prediction accuracy of the signature and the areas under the curves (AUCs) of risk score, age, gender, stage, T, N, M, invasion, mitotane therapy, and radiation therapy were 0.747, 0.538, 0.659, 0.679, 0.745, 0.455, 0.583, 0.727, 0.689, and 0.402, which indicated superior prediction accuracy of the m6A based signature in survival outcomes (**Figure 5F**).

### The m6A Based Signature Is an Independent Prognostic Factor for ACC

**Figure 6:**
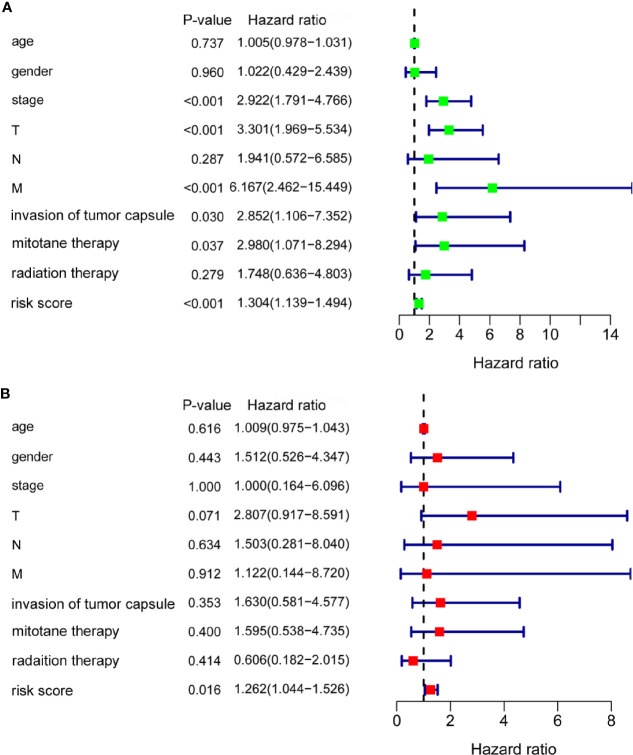
The m6A based signature was an independent prognostic factor for ACC. **(A)** Univariate Cox regression analysis showed that stage, T, M, invasion of tumor capsule, mitotane therapy, and risk score were significantly associated with the OS of ACC patients. **(B)** Multivariate Cox regression analysis demonstrated that the m6A based signature could serve as an independent prognostic factor in ACC.

To evaluate the prognostic significance of the m6A based signature and various clinical factors, univariate and multivariate Cox regression analyses were performed. Univariate Cox regression analysis showed that stage (*p* < 0.001), T (*p* < 0.001), M (*p* < 0.001), invasion of tumor capsule (*p* = 0.030), mitotane therapy (*p* = 0.037), and risk score (*p* < 0.001) were significantly associated with the OS of ACC patients (**Figure 6A**). Multivariate Cox regression analysis demonstrated that the m6A based signature could serve as an independent prognostic factor in ACC (*p* = 0.016, **Figure 6B**).

### Association Between m6A Based Signature Risk Score and Clinical Factors

**Figure 7:**
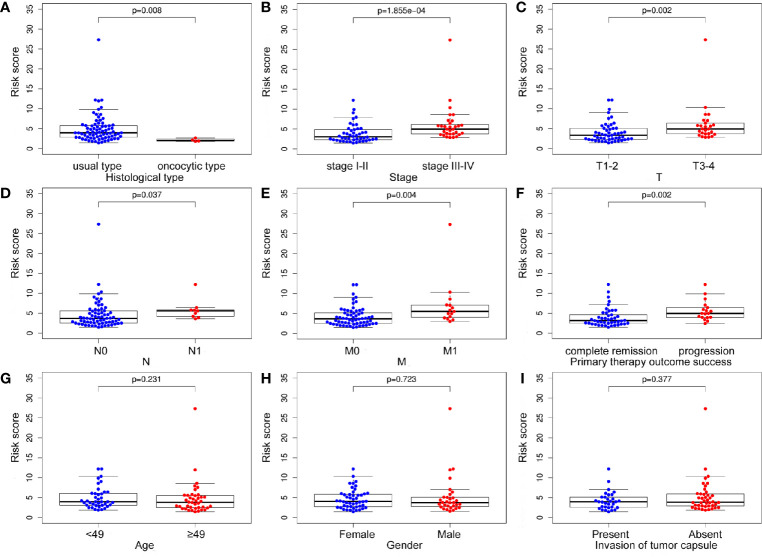
The relationship between the m6A based signature and clinical characteristics. **(A-F)** The risk score was closely related to histological type, stage, T, N, M, and progression of ACC. **(G-I)** However, the signature was not associated with age, gender, and invasion of tumor capsule in ACC patients.

The treatment strategies for ACC patients depend largely on clinicopathologic characteristics. Therefore, we analyzed the relationship between the m6A based signature risk score and clinical factors in ACC patients. Our study showed that the risk score was closely related to histological type (*p* = 0.008), stage (*p* = 1.855e-04), T (*p* = 0.002), N (*p* = 0.037), M (*p* = 0.004), and progression (*p* = 0.002), but not associated with age (*p* = 0.231), gender (*p* = 0.723), and invasion of tumor capsule (*p* = 0.377) in ACC patients (**Figure 7**).

### Construction of a Prognostic Nomogram for ACC

**Figure 8:**
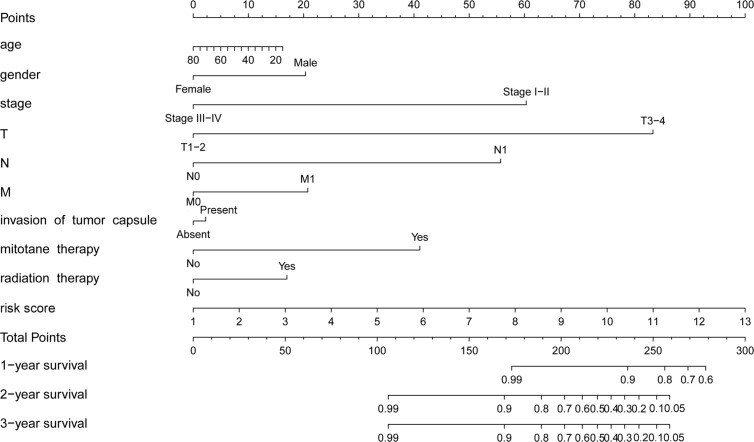
Prognostic nomogram with clinical features and m6A based signature for ACC. The nomogram could superiorly predict 1-, 2-, and 3-year OS of ACC patients.

To establish a clinically applicable method for monitoring the prognosis of ACC patients, we constructed a prognostic nomogram by combining clinical factors (age, gender, stage, T, N, M, invasion of tumor capsule, mitotane therapy, and radiation therapy) with the m6A based signature risk score. The result indicated that the prognostic nomogram could superiorly predict the 1-, 2-, and 3-year OS of ACC patients (**Figure 8**).

### Validation of the Expression Levels of Three m6A RNA Methylation Regulators by *in Vitro* Experiments

**Figure 9:**
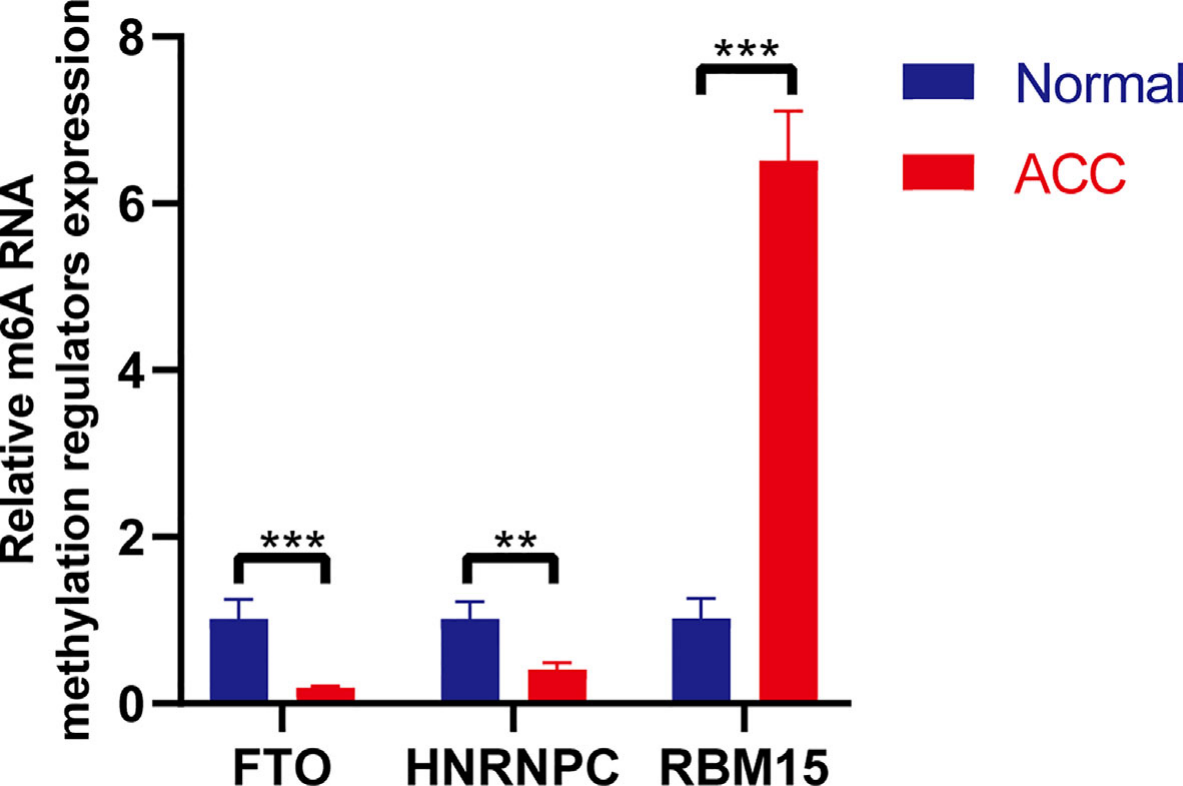
RT-PCR validation of three m6A RNA regulators in ACC and normal tissues. The results indicated that significant differences in the expression levels of three m6A RNA methylation regulators (*RBM15*, *HNRNPC*, and *FTO*) between ACC and normal tissues. **p < 0.01; ***p < 0.005.

RNA isolation and reverse transcription−quantitative PCR (RT-qPCR) were further performed to validate the expression levels of the three selected m6A RNA methylation regulators in 3 newly diagnosed ACC patients. Retroperitoneal laparoscopic resections of the adrenal tumor were performed on all patients. We applied the paired t-test to evaluate the differences between the ACC tissues and the adjacent non-tumor adrenal tissues. The results demonstrated significant differences in the expression levels of three m6A RNA methylation regulators (*RBM15*, *HNRNPC*, and *FTO*) between ACC and normal tissues (**Figure 9**). Compared to normal samples, *HNRNPC* and *FTO* were down-regulated and *RBM15* was up-regulated in ACC samples. The results from this qRT-PCR validation in three newly diagnosed ACC patients were consistent with the above bioinformatics results.

## Discussion

ACC is a rare malignant tumor in humans, and its pathogenesis is a complex process, involving a great number of aberrant gene expression profiles in multiple signaling pathways. There are no effective treatments for ACC patients with advanced stage or metastasis and lack of robust biomarkers to predict the prognosis and metastasis of ACC patients. Considering the importance of m6A RNA methylation regulators in cancer progression, it is crucial to explore biomarkers of prevention and treatment to ACC from m6A RNA regulators. In the present study, we first systematically evaluated the expression levels of 13 m6A RNA methylation regulators and further constructed an m6A based prognostic signature in ACC by using the TCGA database. 11 m6A RNA methylation regulators were differentially expressed in ACC, and *RBM15*, *YTHDF2*, and *HNRNPC* were associated with the prognosis of ACC patients. In addition, based on the expression levels of 13 m6A RNA regulators, we classified ACC patients into two groups, clusters 1/2, through consensus cluster analysis. Survival analysis indicated that clusters 1/2 were significantly related to the survival outcomes of ACC patients. Subsequently, three m6A RNA regulators (*RBM15*, *HNRNPC*, and *FTO*) were finally screened out from a signature and survival analysis revealed a positive correlation between high risk scores and poor survival outcomes in ACC. In addition, the m6A based signature was associated with the OS of ACC patients who received mitotane therapy. Therefore, the m6A based signature may be applied as a novel predictive biomarker for ACC patients after mitotane therapy. Univariate and multivariate analyses demonstrated that the m6A based signature was an independent prognostic factor for ACC. Moreover, the m6A based signature risk score was correlated with histological type, advanced stage, and progression of ACC. These results indicated that the m6A based signature may provide a valuable tool for predicting the prognosis and progression of ACC. Furthermore, a nomogram integrated with m6A based signature and clinical features was constructed, which can superiorly monitor the survival of patients with ACC. Finally, RT-qPCR demonstrated significant differences in the expression levels of three m6A RNA methylation regulators (*RBM15*, *HNRNPC*, and *FTO*) between ACC and normal tissues.

Among these m6A RNA methylation regulators, *FTO*, as a genuine demethylase of m6A modification, negatively regulates the expression of its critical target RNAs, such as ASB2 and RARA. FTO plays its major role through reducing the abundance of internal m6A modification, especially in the 3′ untranslated regions (3′-UTRs), which in turn leads to decreased stability of the target mRNA transcripts and influences the occurrence and progression of acute myeloid leukemia (AML) (16). MYC is a direct and functionally essential target of *FTO*, and *FTO* knockdown enhances the expression level of m6A on MYC mRNA (especially at the 5′ UTR and middle exons), leading to mRNA decay and MYC down-regulation, and thereby inhibition of MYC signaling, which plays an important role in the tumor (17). Previous studies showed that the abnormally high expression of FTO was associated with worse outcomes in cancers, such as gastric cancer, endometrial carcinoma, and lung squamous cell carcinoma (18–20). Moreover, *FTO* influences the progression of cancer by regulating multiple signaling pathways, such as FTO-PGC-1 α signaling axis, mediating PKM2 demethylation, impairing the translation efficiency of E2F1 and Myc (21–24). *ALKBH5* also functions as an m6A demethylase, and increases the expression of its key target, FOXM1 (forkhead box M1), to promote the development of cancers by reducing m6A abundance on target mRNA transcripts (especially at the 3′ UTR) (25). *METTL14* was defined as an m6A writer and exerts its oncogenic role through m6A-dependent post-transcriptional regulation of its critical mRNA targets such as MYB and MYC. METTL14 mainly promotes the expression of MYB and MYC by increasing m6A abundance and enhancing the stability of the target mRNA transcripts (26). The biological function of *METTL3* is likely attributed to the promotion of the translation of its mRNA targets, such as MYC, BCL-2, and PTEN, in an m6A-dependent manner (27). Overexpression of *METTL3* significantly promoted tumorigenesis and metastasis of hepatocellular carcinoma, mostly through regulation of SOCS2 expression by an m6qa- and *YTHDF2*-dependent mechanisms (28). *RBM15*, an RNA binding protein, was categorized as the component of writers and its target is the lncRNA XIST, which mediates X-inactivation and gene silencing during development (29). *RBM15*-assisted XIST methylation is necessary for XIST-mediated silencing, thus revealing the first functional role for transcript-specific methylation directed by *RBM15*. *RBM15* was also significantly upregulated in cancers and was associated with the prognosis of cancers, such as gastric cancer and chronic myelogenous leukemia (CML) (30, 31). Furthermore, *RBM15* can modulate the growth, proliferation, cell cycle, and apoptosis of CML cells *via* Notch signaling (31). The fates of m6A-modified mRNAs rely on the functions of distinct proteins that recognize them (i.e., readers), which may affect the stability, splicing, and/or translation of target mRNAs. Dysregulation of m6A modification and the related proteins also contributes to the initiation and progression of cancers. Members of the YT521-B homology (YTH) domain family of proteins, including *YTHDF1*, *YTHDF2*, *YTHDC1*, and *YTHDC2*, have been identified as direct m6A readers. While *YTHDF2* and *YTHDC2* may promote decay of target mRNAs, *YTHDF1* and *YTHDC2* can promote the translation of target mRNAs. Besides, *YTHDC1* likely impacts splicing and nuclear export of target mRNAs (32–38). *HNRNPC*, a regulator in RNA splicing, has been upregulated in various cancers and tumor cell lines, such as glioblastoma, hepatocellular carcinoma, and lung cancer (39–41). *HNRNPC* may induce human lung cancer cell invasion and metastasis by activating the IFN-α-JAK-STAT1 signaling pathway (42). Wu et al. indicated that inhibition of *HNRNPC* can suppress the proliferation and tumor growth by mediating the cascade of interferon responses (43). In our study, a prognostic signature incorporating three m6A RNA regulators was established based on TCGA data. The AUC of the m6A based signature was 0.747, which showed satisfactory accuracy in predicting the prognosis of ACC patients. Our results indicated that the m6A based signature can be used to identify ACC patients at high risk and provide early interventions to improve the prognosis. However, as a retrospective study, our research still has a bias due to heterogeneity, although almost all the clinical factors in ACC cohorts available from the TCGA database have been included. Second, as a bioinformatic study based on a public database, further experimental studies are needed to explore the potential effect and mechanism of these m6A RNA regulators in the development of ACC.

In conclusion, we identified and validated an m6A based signature, which can be used as an independent prognostic signature in predicting the prognosis of ACC patients. Further clinical trials and experimental explorations are needed to validate our observations and mechanisms underlying the prognostic value of m6A RNA methylation regulators in ACC.

## Data Availability Statement

Publicly available datasets were analyzed for this study. These can be found here: https://tcga‐data.nci.nih.gov/tcga/ and https://xenabrowser.net/datapages/.

## Ethics Statement

The studies involving human participants were reviewed and approved by The Affiliated Hospital of Qingdao University. The patients/participants provided their written informed consent to participate in this study.

## Author Contributions

CS: Conceptualization, methodology, and writing—original draft. JL: Data curation. XY and WJ: Supervision. YW: Writing—review and editing. All authors contributed to the article and approved the submitted version.

## Funding

This work was supported by the National Natural Science Foundation of China (Nos. 81972378, 81101932).

## Conflict of Interest

The authors declare that the research was conducted in the absence of any commercial or financial relationships that could be construed as a potential conflict of interest.

## Supplementary Material

Supplementary Figure 1 The violin plot showed the differentially expressed m6A RNA methylation regulators between cluster 1 and cluster 2.
