# Supplementary figures and images for: Development and Validation of an m6A RNA Methylation Regulators-Based Signature for Predicting the Prognosis of Adrenocortical Carcinoma
Source: Front Endocrinol (Lausanne). 2021 Feb 22;12:568397. doi: 10.3389/fendo.2021.568397 (PMC7937949; doi:10.3389/fendo.2021.568397)

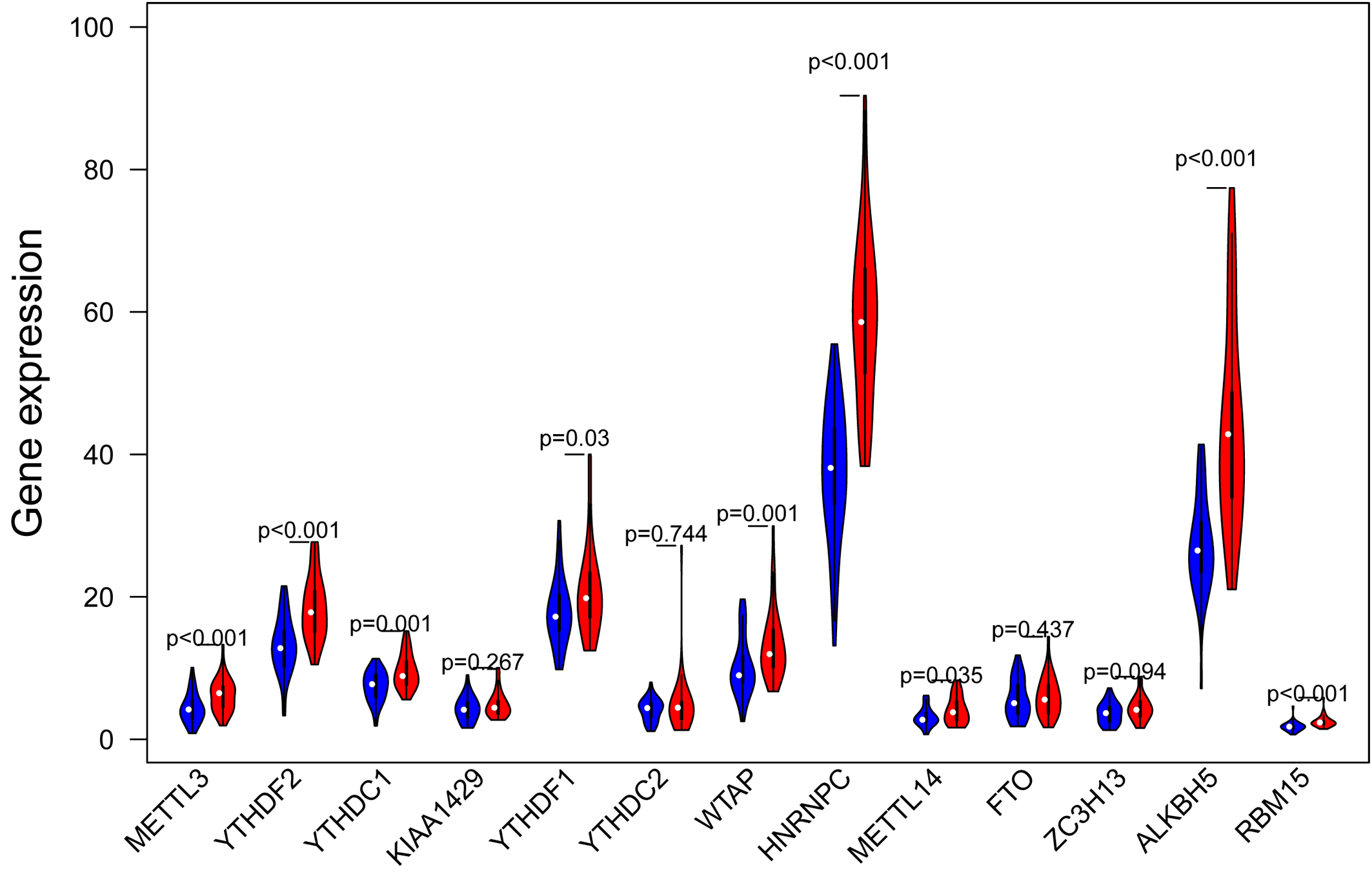

Supplement: Supplementary Figure 1 — The violin plot showed the differentially expressed m6A RNA methylation regulators between cluster 1 and cluster 2. [file Image_1.tif]
