# Supplementary material for: Development and Validation of an m6A RNA Methylation Regulators-Based Signature for Predicting the Prognosis of Adrenocortical Carcinoma
Source: Front Endocrinol (Lausanne). 2021 Feb 22;12:568397. doi: 10.3389/fendo.2021.568397 (PMC7937949; doi:10.3389/fendo.2021.568397)
Supplement: Supplementary file 2 [file Table_1.docx]

The raw data for the QPCR experiment.

| Sample | GADPH | FTO | HNRNPC | RBM15 |
| --- | --- | --- | --- | --- |
| Normal 1 | 15.72 | 22.61 | 20.36 | 26.78 |
| Normal 2 | 15.21 | 22.39 | 20.26 | 26.95 |
| Normal 3 | 14.77 | 21.3 | 19.22 | 25.95 |
| ACC 1 | 12.07 | 21.45 | 18.38 | 20.62 |
| ACC 2 | 12.37 | 21.47 | 18.41 | 20.91 |
| ACC 3 | 13.02 | 22.29 | 18.75 | 21.8 |

The values in the table represent the Cq (∆R) of genes.
